# Preparation of Hierarchical Porous Silicon Carbide Monoliths via Ambient Pressure Drying Sol–Gel Process Followed by High-Temperature Pyrolysis

**DOI:** 10.3390/ma16010220

**Published:** 2022-12-26

**Authors:** Fei Li, Lin Zhou, Ji-Xuan Liu, Guo-Jun Zhang

**Affiliations:** 1 State Key Laboratory for Modification of Chemical Fibers and Polymer Materials, College of Materials Science and Engineering, Institute of Functional Materials, Donghua University, Shanghai 201620, China; 2 Joining and Welding Research Institute, Osaka University, Osaka 5670047, Japan

**Keywords:** silicon carbide, hierarchical porous materials, mechanical properties, sol–gel processing, ambient drying

## Abstract

Hierarchical porous silicon carbide (SiC) attracts great attention due to its superior chemical resistance, high thermal shock resistance, and excellent thermal stability. The preparation of a porous SiC monolith via a simple sol–gel method is limited by either the high cost of the raw materials or the special time-consuming drying process. Herein, we report an ambient drying sol–gel approach for the synthesis of organic–inorganic hybrid monolithic gels which can be converted into hierarchical porous SiC monoliths upon pyrolysis at 1400 °C. The as-synthesized SiC monoliths possess hierarchical pores with macropores of 4.5 µm and mesopores of 2.0 nm. The porosities, specific surface areas and compressive strengths of the hierarchical porous SiC monoliths are 71.3%, 171.5 m^2^/g and 7.0 ± 0.8 MPa, respectively.

## 1. Introduction

Porous solids that are lightweight and composed of air and solid skeletons, possessing excellent specific properties compared to their dense counterparts, have found applications in wide fields, such as use as catalyst supports, thermal insulators, separation and filtration and energy- and environment-related fields, etc. [1,2,3,4,5,6,7,8,9,10,11]. Among the porous solids, macroporous silicon carbide (SiC) materials have attracted great attention in recent decades due to their unique sets of properties including superior chemical resistance, high thermal shock resistance, excellent thermal stability and good mechanical properties [12,13]. Numerous researchers are dedicated to exploring the processing–microstructure–property relationship in macroporous SiC materials [2,14,15,16]. The processing routes for macroporous SiC can be divided into five categories according to Eom’s review: partial sintering, replica, sacrificial template, direct foaming and bonding techniques [12]. During the processing of macroporous SiC materials, sintering at temperatures as high as 1500–2000 °C is generally needed when SiC powders are used as raw materials, while this is avoidable by using preceramic polymers or organic–inorganic hybrid sols containing carbon and silicon as precursors instead [2,12]. By taking advantage of their liquid state, preceramic polymers with tailor-made compositions can be shaped to produce thin films, fibers, coatings or monoliths [17]. Numerous reports focusing on the processing of preceramic polymers to porous SiC-based materials through direct foaming and templating routes can be accessed [2]. The high cost of the preceramic polymers, the toxicity of the reagents and the time-consuming synthesis process are the main drawbacks that limit the wide application of polymer-derived porous SiC materials [15,17,18]. Sol–gel methods involving the hydrolysis and polycondensation of economical carbon-containing monomers and silicon alkoxides followed by pyrolysis are extensively studied as alternative routes [19,20]. Shrinking and cracking are common phenomena during sol–gel transitions, which makes it difficult to obtain bulk SiC materials through the sol–gel method [1]. Expensive special drying techniques (e.g., supercritical fluid drying and freeze drying) and longtime solvent exchange procedures are used to avoid the collapse of the bulk gels [19,21]. 

In this work, the synthesis of monolithic hybrid gels that can be converted into hierarchical porous SiC monoliths upon pyrolysis via a simple ambient drying sol–gel process is reported. Cost-effective furfuryl alcohol (FA), tetraethyl orthosilicate (TEOS) and sodium dodecyl sulfate are used as the carbon-containing monomers, silicon source and surfactant, respectively. Monomers of FA undergo cationic polymerization, and TEOS undergoes hydrolysis with the existence of acidic catalysts to form organic–inorganic hybrid sols [22]. The hybrid sols can be directly dried under ambient pressure to obtain monolithic gels. The as-synthesized hierarchical porous SiC monoliths exhibit excellent mechanical properties.

## 2. Materials and Methods

**Figure 1:**
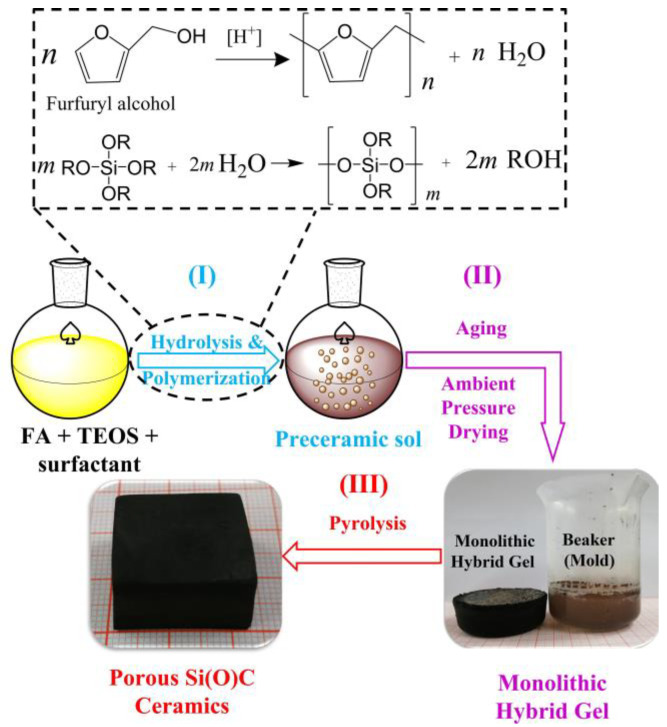
Schematic illustration for the preparation of porous SiC-based materials via sol–gel method.

Furfuryl alcohol (FA, 98%), tetraethyl orthosilicate (TEOS), sodium dodecyl sulfate (SDS), boric acid (BA, 98%), polyethylene glycol (PEG, Mn = 20 000), absolute ethanol and hydrochloric acid (HCl, 1M) were obtained from Sinoharm Chemical Reagent Co., Shanghai, China, and were used as the raw materials. TEOS, BA and PEG were dissolved in ethanol and were mechanically stirred at 50 °C in a water bath for 30 min. FA and 1 M HCl were added dropwise in sequence into the mixture. The mole ratio of TEOS: BA: FA was 1: 2: 2. The mixture was stirred at 60 °C in a water bath for 50 min. After that, SDS was added into the mixture and was stirred for another 10 min to obtain the preceramic sol. The weight fractions of SDS to the mixture were 0, 0.5% and 1.0%. The preceramic sol was dried at 70 °C in an oven for 2 days, which underwent sol–gel transition to obtain a wet gel and finally a dried gel. The dried gels were heated at temperatures ranging from 1100 °C to 1400 °C for 60 min in vacuum. The heating rate was 10 °C/min. The samples (sols, gels and corresponding ceramic products) were denoted as SCB-S0, SCB-S0.5 and SCB-S1 according to the SDS concentration used in the starting compositions. The synthesis procedure is schematically illustrated in Figure 1.

The density, porosity and pore size distribution of the samples were obtained by using a Mercury Porosimeter (MIP, AutoPore IV 9500, Micromeritics, Norcross, GA, USA). Pictures of the hybrid gels were collected by using a digital camera (Nikon 1 J5 with 5568 × 3712 pixel). The colloidal particle size of the preceramic sols and hybrid gels were measured using a Nanometer Particle Size and Potential Analyzer (Malvern Nano ZS/Nano ZS, Great Malvern, UK). The phase compositions of the pyrolyzed samples were analyzed by using a Rigaku D/Max-2200 PC X-ray diffractometer with a Cu target (40 kV, 40 mA). Scanning electron micrographs (SEMs) were taken on a Hitachi SU8200 electron microscope. The specific surface areas and mesopore distributions of the samples were analyzed using a nitrogen sorption analyzer (Autosorb IQ, Quantachrome Instruments, Boynton Beach, FL, USA). The compressive strengths of the SiC ceramic monoliths were tested on a universal testing machine (Instron 5592, Norwood, MA, USA) with a crosshead speed of 0.5 mm/min. The samples were 10 mm × 10 mm × 10 mm in size. Thermogravimetric analysis was performed on a TGA/DSC calorimeter (TGA/DSC 3+/1600 HT, Mettler-Toledo, Zurich, Switzerland) in flowing argon with a heating rate of 10 °C/min. Fourier transform infrared spectra (FT-IR) were collected by using a Thermo Nicolet 6700 spectrometer via the KBr disc method.

## 3. Results and Discussion

**Figure 2:**
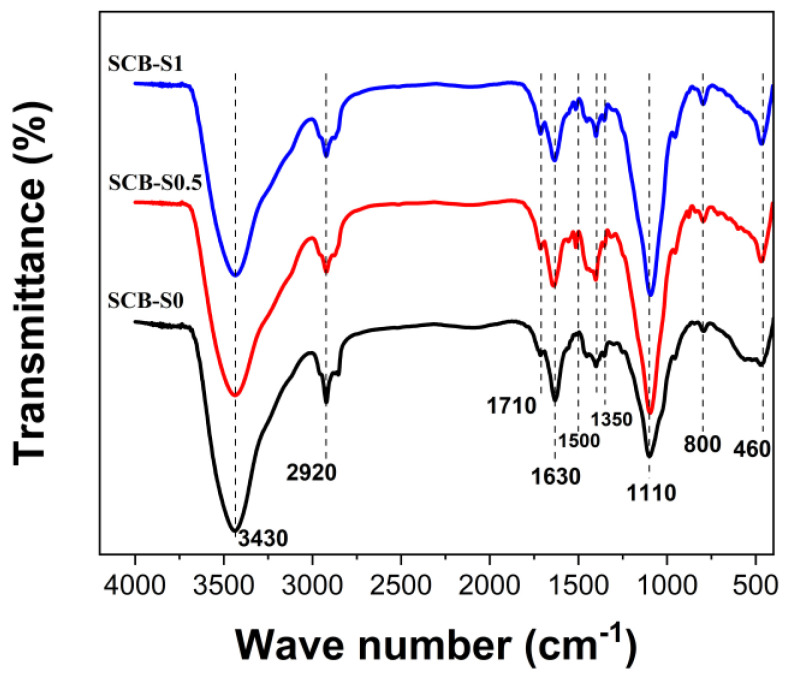
FT-IR spectra of the hybrid gels, TEOS and FA.

During ambient pressure drying, evaporation creates a liquid vapor meniscus at the exit of pores in the gel. This liquid vapor meniscus induces hydrostatic tension in the liquid, which is proportional to the liquid–solid tension and inversely proportional to the pore size. Such hydrostatic tension is balanced by compression on the solid gel networks, which makes the gel network shrink [23]. Cracking may occur when the tension is so large that it cannot shrink anymore. It is essential to decrease the effect of capillary stress for a given structure to obtain monolithic materials [1]. In our reaction system, hybrid gels for porous SiC monoliths were synthesized via the sol-gel method based on the polymerization of FA and hydrolysis of TEOS, using SDS as the surfactant. As a Lewis acid, boric acid could partially catalyze the polymerization of FA [24]. Figure 2 shows the FT-IR spectra of the dried gels. All the bands of the three hybrid gels with different SDS concentrations were similar to each other. The broad band at 3430 cm^−1^ was due to the vibrations of hydroxy groups [25]. The band near 2920 cm^−1^ was ascribed to the C-H vibrations [25]. The absorption band at ~1710 cm^−1^ in the dried gels was attributed to the carbonylic structures, which was considered as a characteristic absorption band for the polymerization of furfuryl alcohol [25]. The absorption band near 1630 cm^−1^ was probably due to the ring stretch modes of the furan rings [25]. The broad absorption band in the range 1300~1500 cm^−1^ could be assigned as the overlapping bands of CH_3_, the furan ring and B-O bonding [25]. The band at ~1100 cm^−1^ in the hybrid gels was assigned to the vibrations of Si-O-Si bonds [26]. The absorption bands at low wave number ranges were ascribed to oxygen bonding with inorganic components (i.e., Si-O and B-O) [7]. 

**Figure 3:**
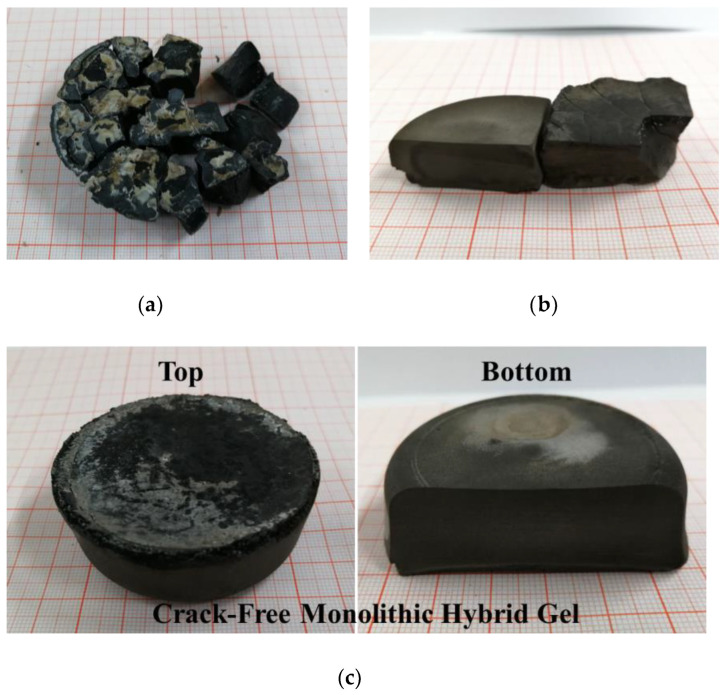
Photographs of the hybrid gels with various SDS concentrations in the starting sols: (**a**) SCB-S0; (**b**) SCB-S0.5; (**c**) SCB-S1.

Figure 3 shows the photographs of the dried gels with various SDS concentrations in the starting sols. Without the use of SDS, the dried gel cracked into fragments during ambient drying, as shown in Figure 3a. Increasing the SDS concentration to 0.5 wt% (SCB-S0.5), fragments with relatively large sizes could be obtained (Figure 3b). A monolithic gel with a diameter over 5 cm was prepared when the SDS concentration was 1.0 wt% in the starting sol (SCB-S1), as shown in Figure 3c. This monolithic gel was crack-free and demonstrated a fine structure by the naked eye. The densities of the dried hybrid gels were 1.24 g/cm^3^, 1.02 g/cm^3^ and 0.62 g/cm^3^, while the porosities were 15.3%, 29.3% and 54.9% for the SCB-S0, SCB-S0.5 and SCB-S1 dried gels, respectively. 

**Figure 4:**
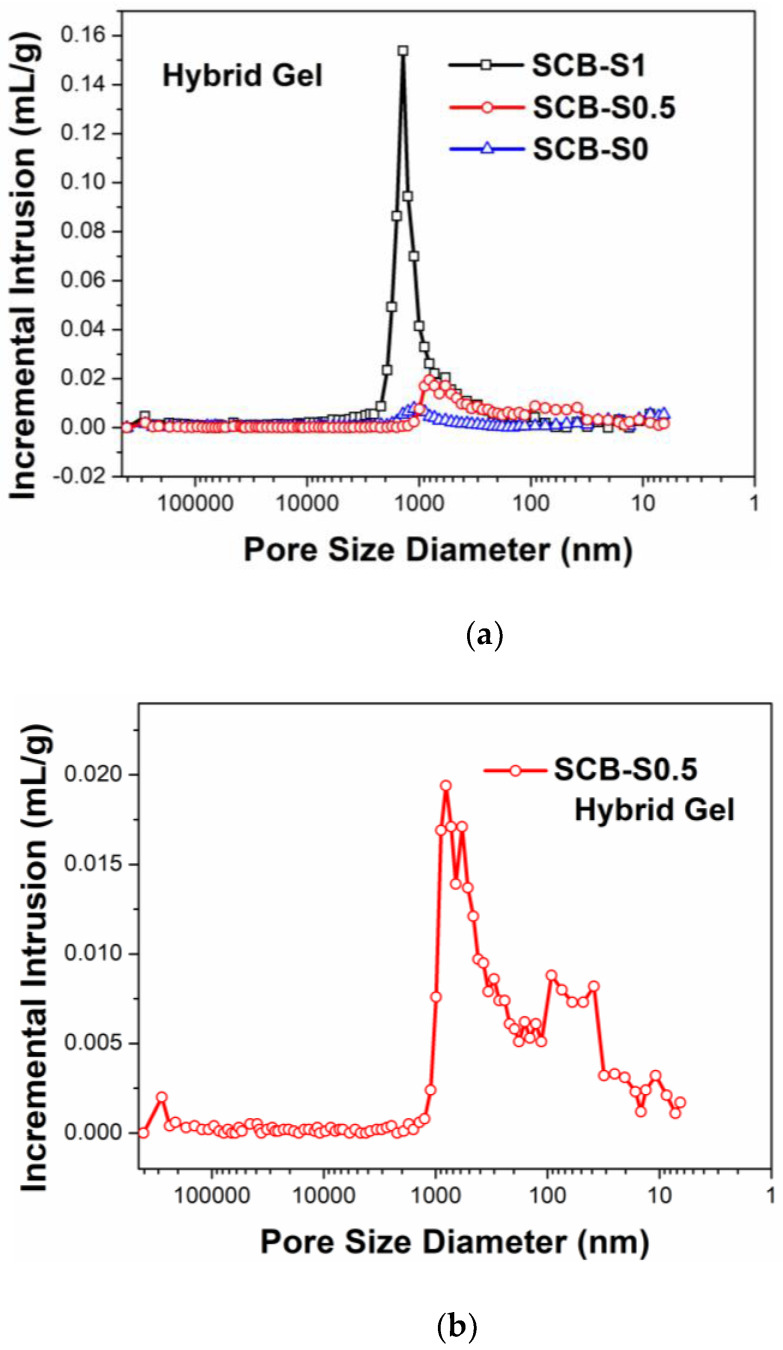

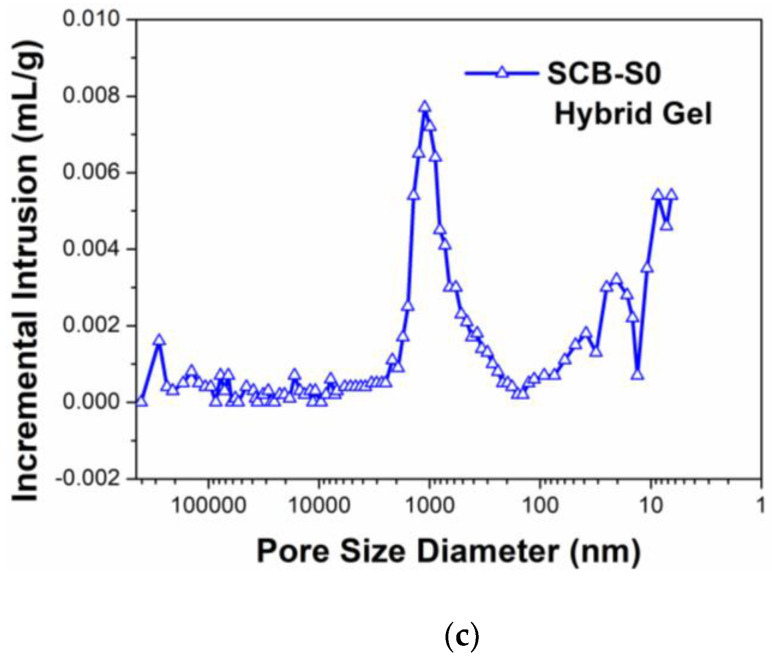
(**a**) Pore size distribution curves of the hybrid gels. Expanded view of (**b**) SCB-S0.5 and (**c**) SCB-S0 curves.

The colloidal particle sizes of the preceramic sols with various SDS concentrations were 237 ± 2 nm, 478 ± 6 nm and 570 ± 11 nm, respectively. Both mesopores and macropores were observed in the pore size distribution curves of the dried hybrid gels, as shown in Figure 4. The hybrid gels of SCB-S0 and SCB-S0.5 demonstrated poor macroporous structures with pore sizes ranging from 100 nm to 2 µm. Hybrid gel SCB-S1 possessed fine macroporous structures with a monomodal pore size distribution peak ranging from 100 nm to 3 µm. The addition of SDS in the preceramic sol led to colloidal particle coarsening (from 237 to 570 nm), which may have been due to the breakup of the electrostatic balance in the original colloid cluster. Coarsened colloids aggregated to construct a gel network with larger pore sizes, which benefitted the decrease in capillary forces during drying [23]. Hence, the shrinking and cracking of the hybrid gels were partially suppressed, and an ambient pressure-dried crack-free monolith with relatively high porosity was successfully prepared. 

**Figure 5:**
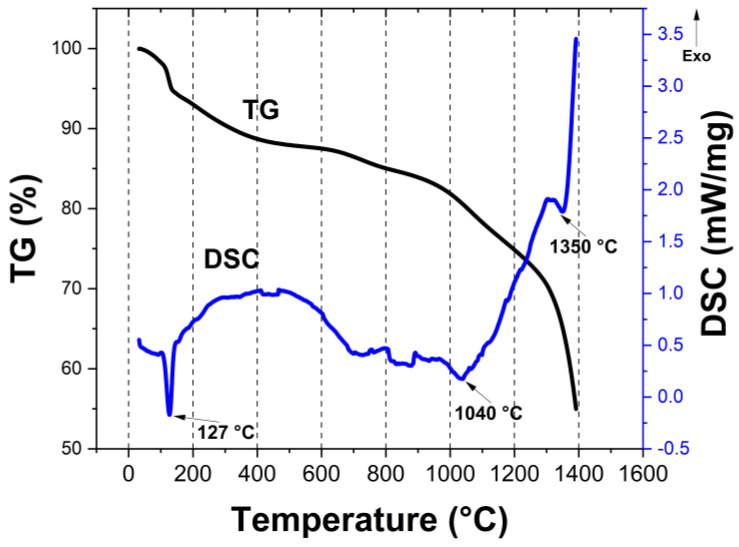
TG-DSC curves of the SCB-S1 hybrid gel under flowing argon.

Figure 5 shows the TG-DSC curves of the SCB-S1 hybrid gel under flowing argon. The TG curve shows that there was a 10 wt% loss at temperatures below 160 °C, accompanied with an endothermic peak in the DSC curve, which was possibly due to the dehydration of the sample. Gradual weight loss at temperatures between 160 °C and 1000 °C in the TG curve demonstrated the decomposition of the organic/inorganic hybrid gels, PEG and SDS. PEG decomposed completely upon heating and had no carbon residue [6]. The weight loss accelerated at >1000 °C, indicating the carbothermal reduction reaction between amorphous carbon and oxides (or oxycarbides). At temperatures above 1200 °C, an almost vertical weight loss curve was observed, demonstrating the rapid carbothermal reduction reaction and the evaporation of volatile boron-containing species [27]. TG-DSC analysis suggested that 1300–1400 °C should have been high enough to complete the carbothermal reduction reaction. The bulk material showed linear shrinkage of 33% during the pyrolysis process, which corresponded to nearly 70% volume shrinkage. 

**Figure 6:**
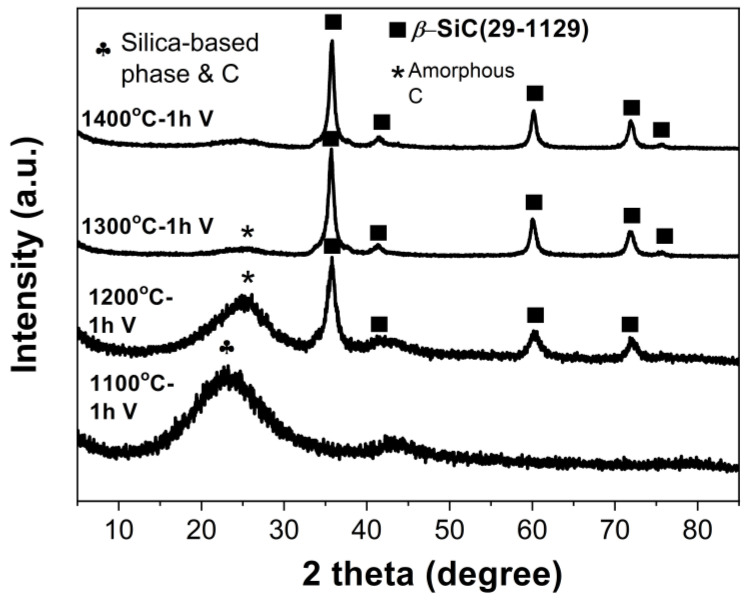
XRD patterns of the hybrid gels after being heated at 1100 °C–1400 °C in vacuum. The holding time at targeted temperature is 1 h.

Figure 6 shows the XRD patterns of the samples pyrolyzed at 1100 °C–1400 °C in vacuum. Only broad diffraction peaks of amorphous phases were detected when the heating temperature increased to 1100 °C. The broad peak at around 22° could be assigned to mixtures of silica-based phase and amorphous carbon. When heated at 1200 °C, it shifted toward a higher diffraction angle, and this could suggest that the silica-based phase was consumed and the amorphous carbon reflection was now predominant. The *ß*-SiC phase became the dominant phase after pyrolysis at 1200 °C, while considerable amorphous carbon could still be detected. The formation of *ß*-SiC was due to the carbothermal reduction reactions between oxides and carbon according to reactions (1)~(2) [12,28]. The crystallinity of *ß*-SiC phase increased with the increase in the pyrolysis temperature. After being heated at 1400 °C for 1 h, the XRD pattern was similar to the one pyrolyzed at 1300 °C. Although a relatively high amount of boric acid was used in the preceramic sol synthesis process, no diffraction peaks related to boron-containing species could be detected in the XRD patterns of the pyrolyzed samples. This phenomenon might have been due to the boron loss during vacuum pyrolysis [27], which warrants further investigation.

SiO_2_ + C = SiO(*g*) + CO(*g*) (1)

SiO(*g*) + C = SiC + CO(*g*) (2)

**Figure 7:**
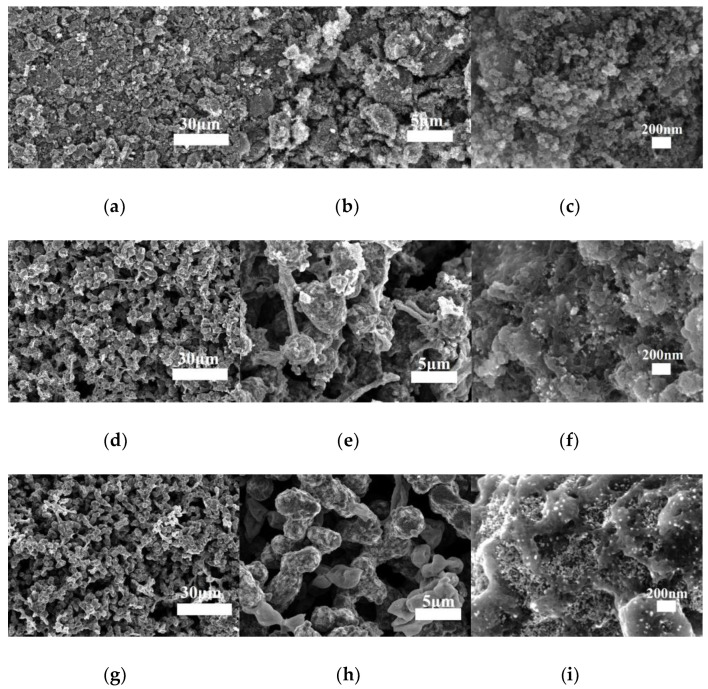
SEM images of the ceramic monoliths after pyrolysis in vacuum at 1400 °C for 1 h. (**a**–**c**): SCB-S0; (**d**–**f**): SCB-S0.5; (**g**–**i**): SCB-S1.

Figure 7 shows the SEM images of the ceramic products after pyrolysis in vacuum at 1400 °C for 1 h. In the case of the SCB-S0 sample, the pyrolyzed product showed a relatively compact morphology. Few pores could be observed, as shown in Figure 7a–c. The porosity, bulk density, and average pore size of the SCB-S0 pyrolyzed products were 34.6%, 1.25 g/cm^3^ and 0.39 µm, respectively, as listed in Table 1. The formation of such a poor porous structure could be ascribed to the large shrinkage and collapse of the gel networks during ambient pressure drying [2]. In the cases of the SCB-S0.5 and SCB-S1 pyrolyzed products, in which SDS was used as a surfactant in the starting sols, the pyrolyzed products possessed highly porous microstructures, as clearly shown in Figure 7d–i. The porous networks were composed of chains of spherical colloidal particles with diameters of about 1–5 µm. The average pore size of the ceramic products was 3.06 µm and 4.50 µm for the SCB-S0.5 and SCB-S1 ceramic monoliths, respectively. The microstructures of the spherical colloidal particles in the SCB-S0.5 and SCB-S1 ceramic products were similar to each other, as they were both composed of nano-sized particles, and intragranular pores could be observed, as shown in Figure 7f,i. A higher SDS concentration in the starting sols led to higher porosity and lower density in the final ceramic products, as listed in Table 1. 

**Figure 8:**
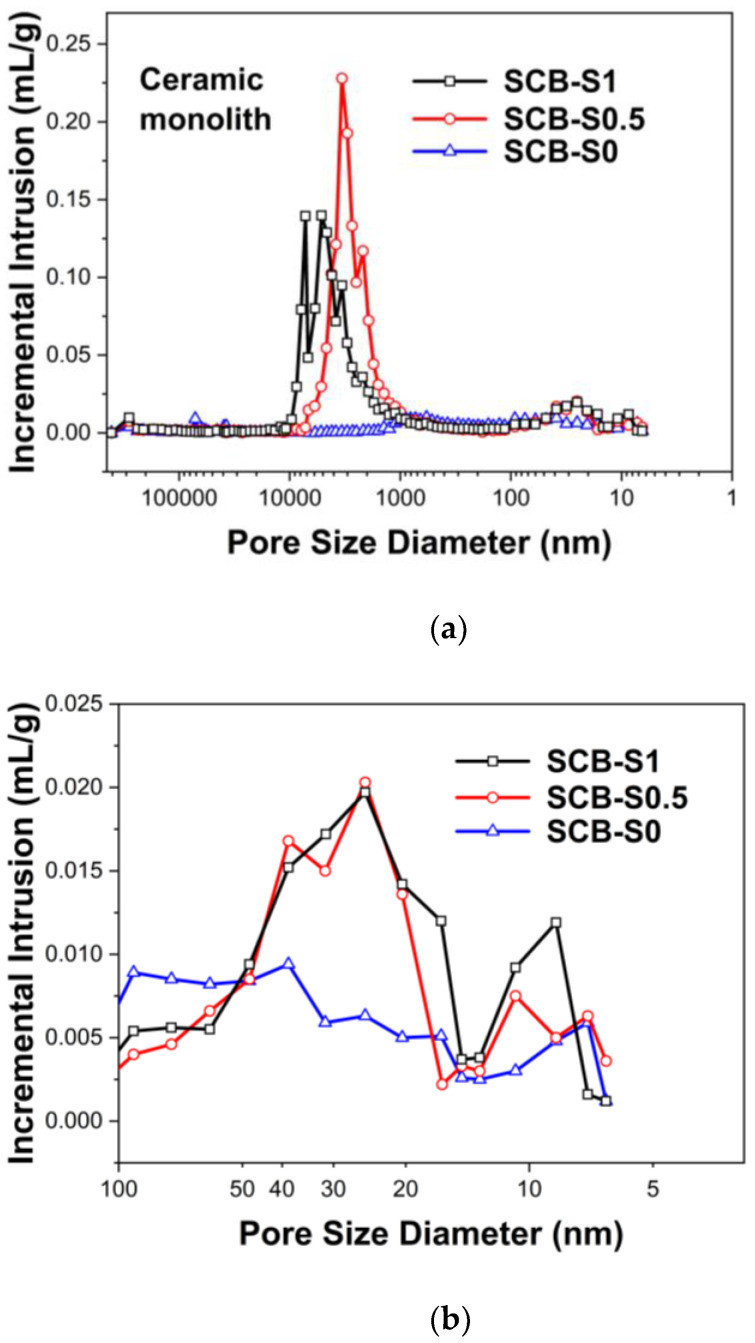

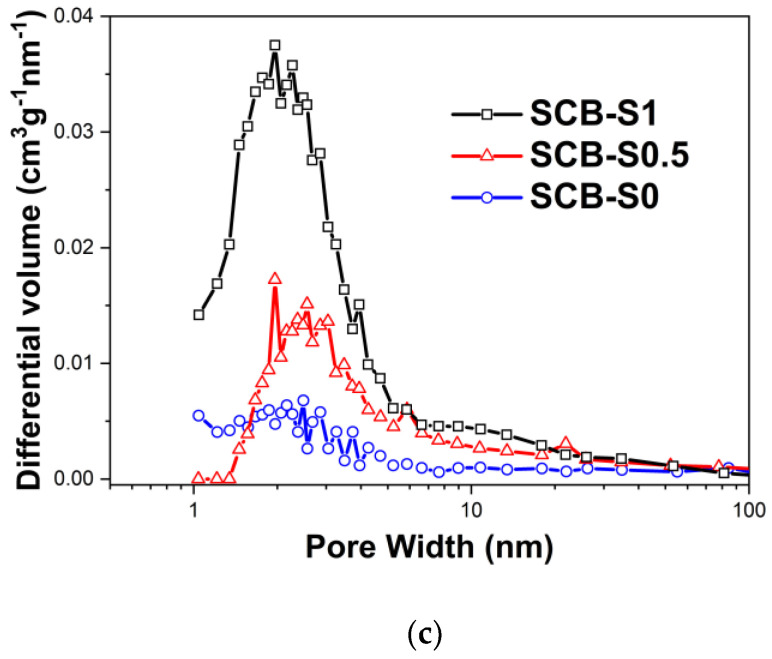
(**a**) Pore size distribution of the porous SiC monoliths after being heated at 1400 °C in vacuum for 1 h. (**b**) Expanded view of the pore size distribution curves in the nanometer scale. (**c**) Pore size distribution of the porous SiC monoliths obtained via nitrogen sorption method.

The pore size distribution curves of the ceramic monoliths both measured via MIP and nitrogen sorption are shown in Figure 8. In Figure 8a, the macropore size distribution curve of the SCB-S0 sample confirms its poor macroporous structures, which is highly consistent with the SEM observations (Figure 7a–c). In the cases of the SCB-S0.5 and SCB-S1 samples, their pore size distribution curves indicate hierarchical porous structures. Strong pore size distribution peaks in the range from 1 µm to 10 µm and weak peaks within 5–100 nm could be observed. A large amount of mass losses and volume shrinkage (~60 vol%) occurred during high-temperature pyrolysis (Figure 5) [2,28]. Comparing Figure 4 and Figure 8a, the pyrolysis process resulted in the increase in the pore sizes at the micron scale. It is interesting to note that the pore size distribution curves of SCB-S0.5 and SCB-S1 at the nanometer scale were quite similar to each other, while almost no peaks could be seen in the SCB-S0 ceramic sample, as shown in the inset of Figure 8a and in Figure 8b. The mesopore size distribution peak intensity increased with the SDS concentration, and so did the specific surface area (as listed in Table 1). Such results imply that SDS plays a key role in maintaining mesopores during drying, which can also survive during high-temperature pyrolysis. Nano-sized particles aggregate to form micro-sized clusters first, and then are constructed into three-dimensional interconnected macroporous networks [6]. 

**Figure 9:**
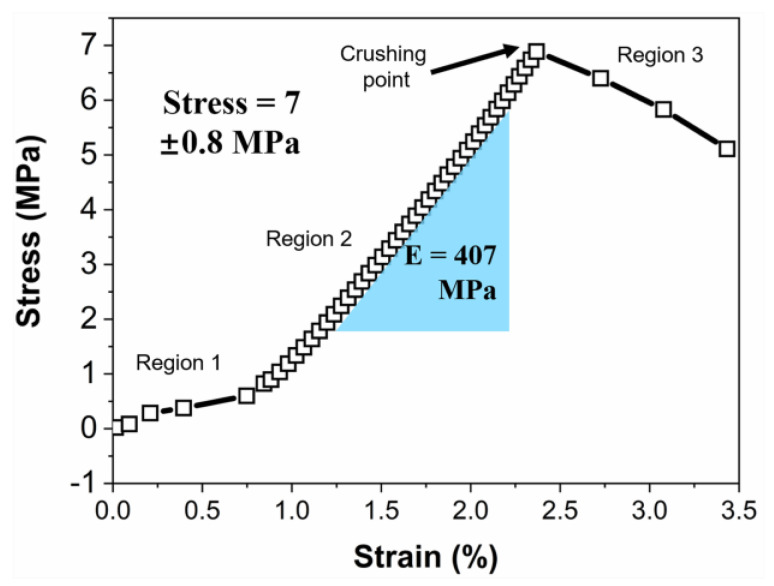
Compressive strain–stress curve of the SCB-S1 ceramic monolith.

Mechanical strength is one of the important features that influence the application of porous solids. Figure 9 shows a typical strain–stress plot of the SCB-S1 monolith under uniaxial compression. The strain–stress could be categorized into three regions. Region 1 could be ascribed to the fixture alignment between the specimen and loading head [29]. In Region 2, a linear elastic strain–stress response was observed. The hierarchical porous monolith could involve a skeleton network fracture and recover to its original shape once the loading is removed. The Young’s modulus was calculated as the slope of strain–stress curve in Region 2, and its value was 407 MPa. The stress continued to rise as the load increased and reached up to the crushing point. At this point, macroscopic cracks propagated through the monolith, leading to the sharp drops in the strain–stress curve (Region 3). The hierarchical porous SiC monolith showed typical brittle behavior. Its compressive strength was 7.0 ± 0.8 MPa. Such relatively high compressive strength possibly originated from its three-dimensional interconnected solid networks. 

## 4. Conclusions

In summary, organic–inorganic monolithic gels that could be converted into SiC hierarchical porous materials upon pyrolysis were prepared via a simple ambient drying sol-gel process with the help of SDS. SDS could lead to colloidal particle coarsening in the hybrid sol and could construct three-dimensional solid networks with larger pore sizes, which benefitted the decrease in capillary pressure, which causes cracking. The monolithic hybrid gels were converted into *ß*-SiC when heated at 1400 °C. The porosity and pore size of the SiC macroporous monoliths could be tailored by tunning the SDS concentration used in the hybrid sols. The specific surface area of the SiC monoliths could reach up to 171.5 m^2^/g. The compressive strength and Young’s modulus of the SiC monoliths were 7.0 ± 0.8 MPa and 407 MPa, respectively. 

## Figures and Tables

**Table 1:** Pore size, bulk density, porosity and specific surface area of the hierarchical porous SiC monoliths.

Ceramic Monoliths	Macropore Size ^1^ (µm)	Mesopore Size ^2^ (nm)	Bulk Density (g/cm^3^)	Porosity (%)	Specific Surface Area (m^2^/g)	
					^1^	^2^
SCB-S0	0.39	2.5	1.25	34.6	15.9	30.5
SCB-S0.5	3.06	2.0	0.72	61.5	24.6	65.0
SCB-S1	4.50	2.0	0.42	71.3	26.5	145.0

^1^ Calculated from MIP data. ^2^ Calculated from nitrogen sorption data.

## Data Availability

Not applicable.
